# Supplementary material for: Manduca sexta experience high parasitoid pressures in the field but minor fitness costs of consuming plant secondary compounds
Source: Ecol Evol. 2021 Sep 9;11(20):13884–97. doi: 10.1002/ece3.8094 (PMC8525118; doi:10.1002/ece3.8094)
Supplement: Supplementary file 1 — Supplementary Material [file ECE3-11-13884-s001.pdf]

SI Figure 1. Fate of *Manduca sexta* larvae collected from a cultivated field of *Nicotiana tabacum* in Kentucky, USA at three time points. A total of 395 *M. sexta* larvae were collected from the field over the three collection dates (21 July 2013 N=98; 20 August 2013 N=156; 28 July 2014 N=141). The parasitoids emerging from field-collected *M. sexta* were *Cotesia congregata* (“larval parasitoids,” black band) and Tachinid flies (“pupal parasitoids,” small dark gray band present in July 2013 and July 2014). *Manduca sexta* larvae that did not survive but did not show external evidence of parasitoids are indicated by the light gray band. The larvae that survived and pupated as adults in the lab (white band) were mated and used to generate the experimental field-collected colony in the greenhouse.

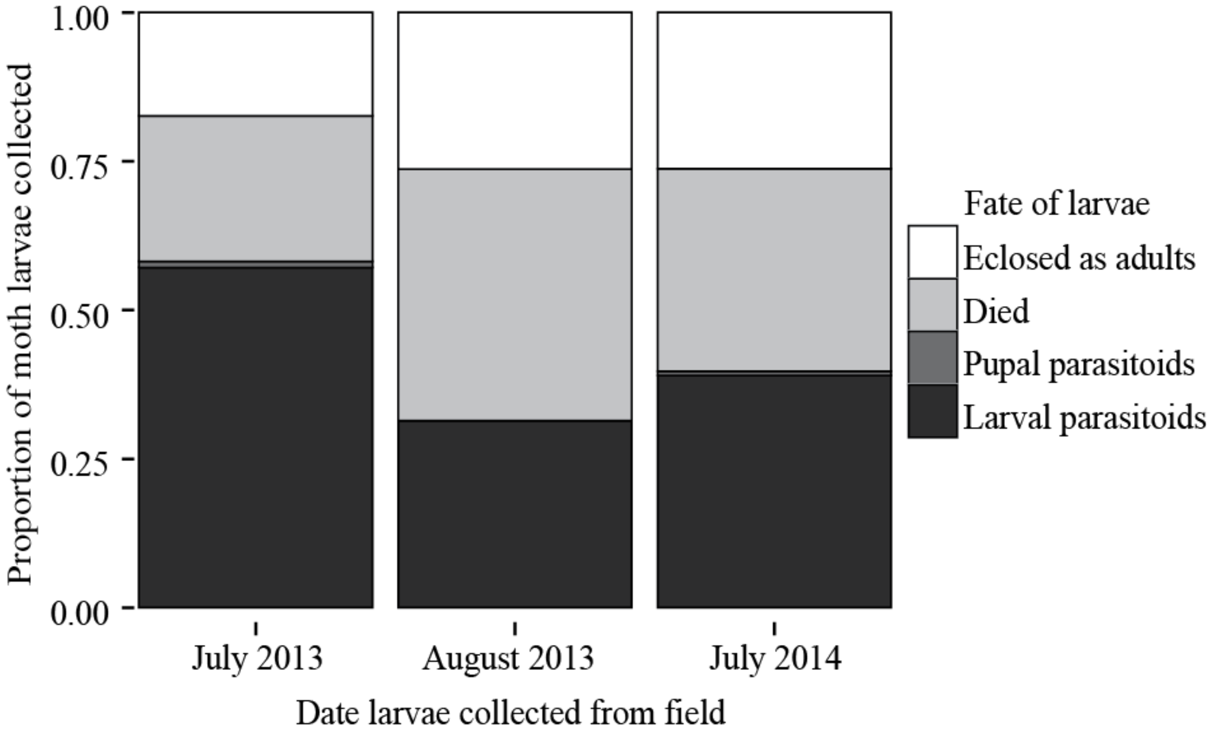

Supplementary methods 1: Establishment of a field-collected colony

Once surviving field-collected *M. sexta* reached the pupal stage, they were transferred to a large cage to eclose and mate in order to establish a field-collected colony to use for the experiments testing the effects of secondary compounds on *M. sexta* developmental and fitness. Pupae were placed over a tray of wet gravel in a greenhouse with 14D:10N light cycles and misted daily with water to increase humidity in the cage. The cage was draped with a sheet to keep the moths cool and increase darkness at night. Adult moths were provided with a sponge saturated with 20% honey water for nectaring (Hunter's honey farm, Martinsville IN, USA) and a Solanaceous host plant for egg deposition. Bergamot oil was also applied to the cage every 1-3 days to increase nectaring and stimulate mating (Goyret and Raguso 2006). Eggs produced from the matings were collected and surface sterilized with 1% bleach and rinsed in distilled water. After hatching, *M. sexta* larvae were reared individually in two-ounce clear plastic lidded containers until approximately the third instar, when they were transferred to similar lidded four-ounce containers.

Although *M. sexta* larvae directly collected from the field required the addition of leaf tissue to the artificial diet, larvae of subsequent generations in the lab were reared solely on artificial diet (SI Table 1). Approximately 60 percent of the larvae take to the artificial diet while the remaining die within the first two to four days after hatching after failing to accept the artificial diet.

SI Table 1. Recipe for artificial diet used for *Manduca sexta* larval rearing modified from the University of Madison Wisconsin Department of Entomology diet. Experimental and control diets were refrigerated and kept for no longer than one week to prevent degradation of the chemicals over time. For the growth trials, artificial diet was refreshed or replaced every 1-2 days. For the choice trials using neonate larvae, fresh diet was made the day of the trial. To create 0.5% rutin and 0.5% nicotine diets, pure liquid nicotine or powdered rutin were added to diets once cooled but before hardening. The amount of water for control diet (530 mL) was adjusted for the experimental diet to maintain consistency with the addition of 0.5% nicotine (liquid) or 0.5% rutin (powdered). Artificial diet was blended with 10-20 percent fresh leaf tissue for the larvae collected in the field (tobacco in 2013 and *Datura stramonium* in 2014) as larvae will not consume artificial diet lacking plant material after feeding on leaves.

Ingredient	Amount
Water	530 mL*
Agar	20 g
Non-toasted wheat germ	100 g
Non-fat dry milk	22 g
Sugar	13.6 g
Vitamin C (generic)	1 tablet
Vitamin B (generic)	2 tablets
Multivitamin (generic)	2 tablets
Raw flax seed oil	1 tsp
Nutritional flake yeast	3.8 g
Sorbic acid (preservative)	1.5 g
Methyl-4-hydrobenzoate (preservative)	1 g

SI Table 2. Secondary compounds increased the amount of *Manduca sexta* larvae completing an extra (sixth) instar (N = 80 per treatment and colony). Numbers of larvae completing six instars are presented for control and nicotine fed larvae (left) and control and rutin (right) for the field and lab colonies. Significant *P* values (< 0.05) from Fisher's exact tests are indicated with an asterisk (*).

	**control**	**nicotine**	***P***	**control**	**rutin**	***P***
**Field**	5	2	0.44	0	6	0.03*
**Lab**	0	6	0.02*	0	8	<0.01*

SI Table 3. Pairwise comparisons among pupal and adult traits. Separate controls are presented for nicotine and rutin because the effects of these secondary compounds were tested at different generations. Significant *P* values (< 0.05) are indicated with an asterisk (*).

		field control		field 0.5% nicotine		lab control		lab 0.5% nicotine		field control		field 0.5% rutin		lab control		lab 0.5%rutin	
		cor	*P*	cor	*P*	cor	*P*	cor	*P*	cor	*P*	cor	*P*	cor	*P*	cor	*P*
**follicles**	**body length**	0.45	0.08	0.14	0.50	0.04	0.84	-0.10	0.58	0.27	0.51	0.30	0.47	0.08	0.68	0.24	0.24
**follicles**	**pupal wt**	0.53	0.04*	0.18	0.39	0.05	0.79	0.12	0.52	-0.02	0.96	0.41	0.31	0.07	0.72	0.06	0.79
**follicles**	**body area**	0.61	0.01*	0.24	0.24	-0.10	0.59	0.18	0.33	0.25	0.56	0.23	0.58	0.27	0.14	0.23	0.28
**body length**	**pupal wt**	0.59	<0.01*	0.56	<0.01*	0.63	<0.01*	0.59	<0.01*	0.59	<0.01*	0.67	<0.01*	0.49	<0.01*	0.56	<0.01*
**pupal wt**	**body area**	0.67	<0.01*	0.68	<0.01*	0.65	<0.01*	0.69	<0.01*	0.60	<0.01*	0.84	<0.01*	0.68	<0.01*	0.77	<0.01*
